# Tissue Culture Response and In Vitro Plant Regeneration of *Malus* ‘Baiyun’ (a New Cultivar of Ornamental Crabapple)

**DOI:** 10.3390/plants13152080

**Published:** 2024-07-26

**Authors:** Jingze Ma, Junjun Fan, Wangxiang Zhang, Ruomiao Zhou, Yiting Shen, Qin Peng, Huimin Li, Cong Lei

**Affiliations:** 1 College of Forestry, Nanjing Forestry University, Nanjing 210037, China; jingzema@njfu.edu.cn (J.M.); renniezrm@163.com (R.Z.); 13666790461@163.com (Y.S.); penqin@njfu.edu.cn (Q.P.); lihuimin2000@163.com (H.L.); leicong@njfu.edu.cn (C.L.); 2 College of Horticulture, Jinling Institute of Technology, Nanjing 210038, China; 3 Co-Innovation Center for Sustainable Forestry in Southern China, Nanjing Forestry University, Nanjing 210037, China

**Keywords:** *Malus* ‘Baiyun’, tissue culture, ornamental crabapple, shoot proliferation, rooting

## Abstract

*Malus* ‘Baiyun’ (registration no. 20210210), a new crabapple cultivar, was registered in 2021 by the Nanjing Forestry Unversity. However, the difficult rooting has greatly limited the production of high-quality *M*. ‘Baiyun’ in industrialization development. There is thus a pressing need to develop an organogenesis protocol for the in vitro propagation of *M.* ‘Baiyun’ to alleviate a shortage of high-quality *M.* ‘Baiyun’ seedlings. The results showed that choosing the apical bud in mid-March was an excellent explant material. To promote proliferation, the highest proliferation (6.27) of apical shoots was cultured on Murashige and Skoog (MS) medium supplemented with 0.5 mg·L^−1^ 6-benzylaminopurine(6-BA) + 0.05 mg·L^−1^ indole-3-butyric acid (IBA). Subsequently, a 100% rooting rate, average number of roots per shoot of 6.2 and maximum length of roots of 4.96 cm were obtained on half-strength Murashige and Skoog (1/2 MS) medium with the application of 0.5 mg·L^−1^ naphthaleneacetic acid (NAA) or 0.6 mg·L^−1^ NAA + 0.7 mg·L^−1^ IBA. Additionally, thick and lateral roots were obtained with 0.6 mg·L^−1^ NAA + 0.7 mg·L^−1^ IBA. Our study is the first to establish an effective organogenesis protocol for new crabapple cultivars using stem segments.

## 1. Introduction

**Figure 1:**
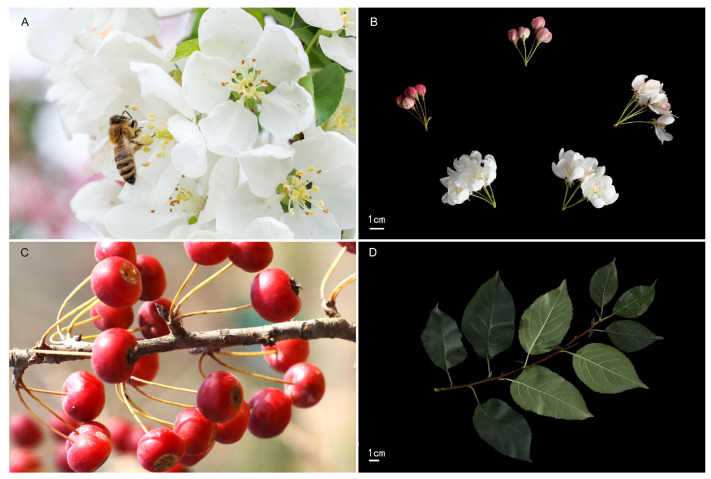
Characteristics of flowers, fruit, and foliage of *Malus* ‘Baiyun’ crabapple. (**A**) Flowers of *M.* ‘Baiyun’; (**B**) the blooming stage of inflorescence in *M.* ‘Baiyun’; (**C**) mature fruit; (**D**) annual branches and leaves.

**Figure 2:**
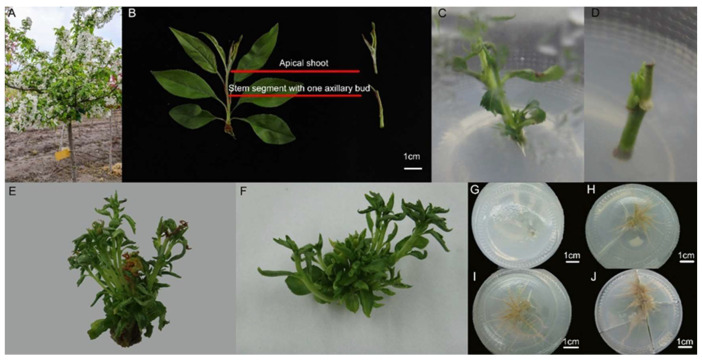
In vitro plant regeneration of *M*. ‘Baiyun’. (**A**) Tree used for propagation (about 2.5 m high); (**B**) different site of explant material; (**C**) proliferation of adventitious shoots (explant: apical shoot); (**D**) proliferation of adventitious shoots (explant: stem segment with one axillary bud); (**E**,**F**) proliferation of *M*. ‘Baiyun’ shoots in different combinations of PGRs 30 d. (**E**) 6-BA + NAA; (**F**) 6-BA + IBA; (**G**–**J**) proliferation of roots of several representative treatments in different PGRs 30 d. on the proliferation of roots. (**G**) PGR-free (CK); (**H**) IBA 1.5 mg·L^−1^ (Treatment No. B6); (**I**) NAA 0.5 mg·L^−1^ (Treatment No. B1); (**J**) NAA 0.6 mg·L^−1^, IBA 0.7 mg·L^−1^ (Treatment No. C3).

The ornamental crabapple (*Malus* spp.) is a deciduous tree or shrub that belongs to the Rosaceae family. It is a general term used for plants with smaller fruits (≤5 cm) in the genus *Malus* and some plants in the genus *Chaenomeles*. The crabapple is an important woody ornamental plant because of its unique characteristics, such as flower, leaf and fruit with excellent ornamental value and delicious fruit [1,2]. *Malus* ‘Baiyun’ (registration no. 20210210) is a new cultivar bred at the National Crabapple Germplasm Genetic Center (Yangzhou City, lat. 32°42′ N, long. 119°55′ E, hardiness zone 8). Its flowers are especially white in spring and its fruits are bright red in autumn and winter. The best ornamental period of the year can last up to 4 months, giving an especially long ornamental period that can be enjoyed in three seasons of the year (Figure 1 and Figure 2A), with good waterlogging resistance. Over the past 10 years, significant progress has been made in breeding new crabapple cultivars. More than 130 new cultivars, including *M.* ‘Baiyun’, have been registered in China. However, at present, *M.* ‘Baiyun’ and other crabapple cultivars, such as *M.* ‘Yanyu Jiangnan’ and *M.* ‘Datangqinhong’ [3,4,5,6], are usually propagated through grafting propagation [7]. Ornamental crabapples can achieve a survival rate exceeding 90% through grafting [3,4]. Grafting can maintain the consistency of ornamental characteristics in new cultivars. However, if resistance is related to the roots, new cultivars of crabapples cannot keep their resistance well due to the influence of rootstocks [8]. Therefore, establishing asexual reproduction methods with roots of new crabapple cultivars is of great significance and urgency to keep all its merits.

Common methods of asexual reproduction with roots include layering propagation, cutting propagation, and plant tissue culture [9]. Layering propagation is used little in woody ornamental plants [10]. The rooting rate of the original species of crabapples is 50.47~83% through cutting propagation [11,12,13]. However, rooting is significantly more challenging for new cultivars. Similarly, *M*. ‘Baiyun’ is confronted with the same challenge.

Therefore, this study aims to establish a tissue culture program with a high rooting rate of *M.* ‘Baiyun’ and solve its challenges of difficult rooting. We analyzed the effects of different basic media, plant growth regulator treatments and other factors on the tissue culture of primary and secondary *M.* ‘Baiyun’ to identify the optimal formula for rooting and establish a set of *M.* ‘Baiyun’ tissue culture technology systems. The established technology system can be a reference for maintaining and promoting good resistance in new cultivars, researching gene function and the factory nursery of *M.* ‘Baiyun’ or other crabapple species.

## 2. Results

### 2.1. Establishing Explants Aseptic System

**Figure 3:**
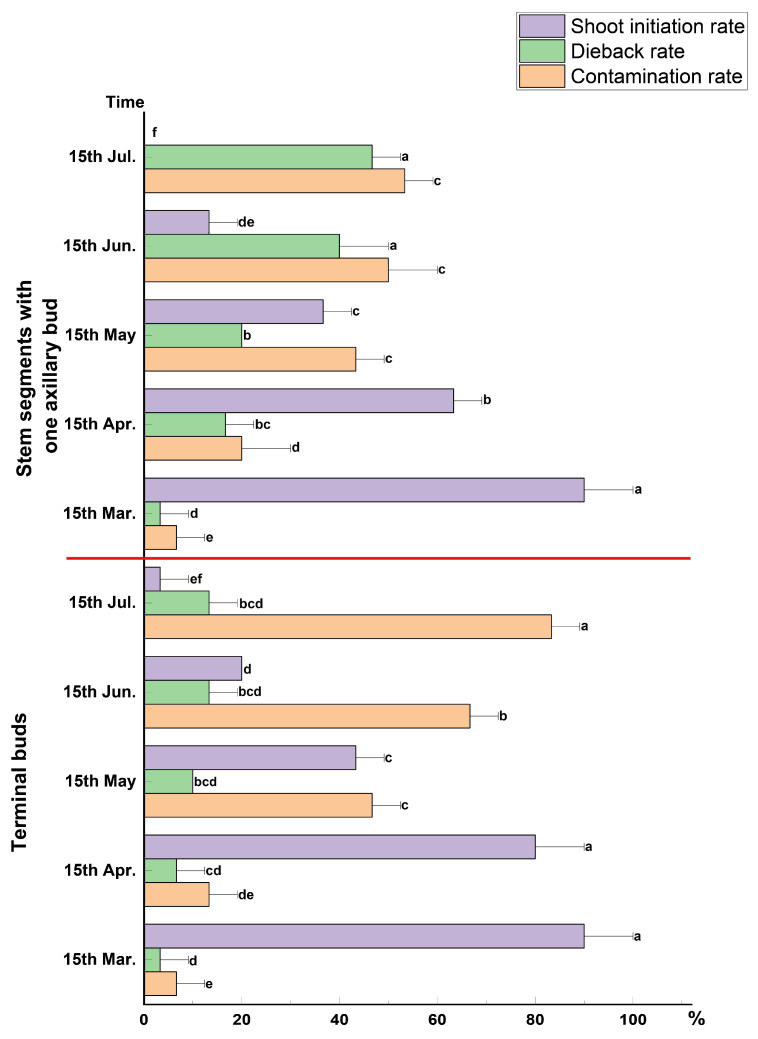
The effect of different extraction sites and times on the sterilization of explants. Two sites: Apical shoots and stem segments with one axillary bud. Five times: 15 March, 15 April, 15 May, 15 June, 15 July. Within each color, mean values with the same letter(s) were not statistically significant based on Duncan’s at *p* ≤ 0.05.

As shown in Figure 3, there were significant differences in the sterilization of the explants depending on the location and the time after the sterilization treatment. After the explants were sterilized by immersion in 75% alcohol (C_2_H_5_OH) for 20 s, within each sampling period, the explants collected in mid-March exhibited the strongest disinfection effect, resulting in the lowest contamination rate and dieback rate of 6.67% and 3.33%, respectively, and the highest shoot initiation rate of 90.00%. However, as the sampling time elapsed, the contamination rates and dieback rates gradually increased while the shoot initiation rates decreased. When using different sampling sites as explant material, selecting terminal buds as explants generally resulted in lower dieback and higher shoot initiation rates than selecting stem segments with one axillary bud. However, the dieback rate was generally poor. Therefore, the period between apical shoots and stem segments with one axillary bud from annual shoots of *M.* ‘Baiyun’ had significant differences in the sterilization effect of the explants. In conclusion, the optimal method for obtaining the material was to choose the terminal bud in mid-March as the explant material.

### 2.2. Effect of Different Treatments with Plant Growth Regulators on the Proliferation of Adventitious Shoots

Table 1 shows the effects of different treatments with plant growth regulators on the proliferation of adventitious shoots. When shoot growth was induced with different plant-growth regulators (6-BA: 6-benzylaminopurine, NAA: Naphthaleneacetic acid, IBA: Indole-3-butyric acid), the combination of 6-BA + IBA caused shoot growth in *M.* ‘Baiyun’. The tissue culture of *M.* ‘Baiyun’ was characterized by a high proliferation coefficient, early and robust axillary shoot and tender and green leaves. Then, the combination of 6-BA + NAA treatment caused some leaf yellowing. Thus, the induction of the combination of 6-BA + IBA shoot proliferation was more effective overall than the 6-BA + NAA combination (Figure 2E,F). In the 6BA + NAA combination treatments, the A5 treatment was significantly better than the other combinations. However, it was significantly lower than the optimal treatment A10 induced by 6BA + IBA. The proliferation effect of the A10 treatment was significantly better than that of other plant growth regulator treatments. The proliferation coefficient of the A10 treatment was 6.27, which was the highest among all treatments. In contrast, the lowest proliferation effect was observed in the A9 treatment, which was 5.5 times lower than that of the A10 treatment (Table 1). The treatment of 0.5 mg·L^−1^ 6-BA + 0.05 mg·L^−1^ IBA plant growth regulator combination was the most effective on the proliferation of adventitious shoots. Therefore, 6-BA + IBA was the excellent combination of PGRs for adventitious shoots induction and the suitable culture formula for *M.* ‘Baiyun’ adventitious shoots induction was MS medium + 30 g·L^−1^ sucrose + 0.5 g·L^−1^ PVP + 7.0 g·L^−1^ agar + 0.5 mg·L^−1^ 6-BA + 0.05 mg·L^−1^ IBA.

### 2.3. Effect of Different Treatments with Plant Growth Regulators on the Rooting

**Figure 4:**
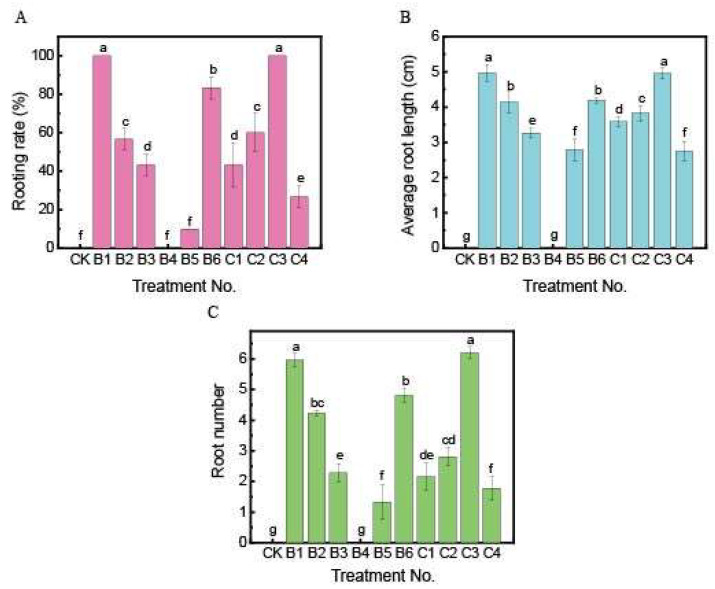
Rooting of *M.* ‘Baiyun’ in different PGRs 30 d. (CK) PGR-free; (B1) NAA 0.5 mg·L^−1^; (B2) NAA 1.0 mg·L^−1^; (B3) NAA 1.5 mg·L^−1^; (B4) IBA 0.5 mg·L^−1^; (B5) IBA 1.0 mg·L^−1^; (B6) IBA 1.5 mg·L^−1^; (C1) NAA 0.4 mg·L^−1^, IBA 0.7 mg·L^−1^; (C2) NAA 0.4 mg·L^−1^, IBA 1.0 mg·L^−1^; (C3) NAA 0.6 mg·L^−1^, IBA 0.7 mg·L^−1^; (C4) NAA 0.6 mg·L^−1^, IBA 1.0 mg·L^−1^. Moreover, for each color, mean values with the same letter(s) were not statistically significant based on Duncan’s at *p* ≤0.05. (**A**) Rooting rate; (**B**) average root length; (**C**) number of roots.

Figure 4 shows the effects of different growth regulator treatments on rooting. Rooting could not be induced without adding exogenous auxin to the 1/2 MS medium (Figure 2G and Figure 4). The addition of single plant-growth regulators (NAA or IBA) had a significant effect on rooting. The B1 treatment was highly significant compared to the other single auxin treatments. The rooting effect of NAA was better than that of IBA, especially the B1 treatment, which achieved a 100% induction rate of rooting (*p* < 0.05) (Figure 2H,I). The B1 treatment resulted in a 100% induced rooting rate, with an average of 5.97 roots and an average root length of 4.97 cm. All rooting indexes were significantly better than those of other single plant growth regulator treatments with different concentrations (*p* < 0.05) (Figure 2H and Figure 4).

The B4 treatment, treated with a single plant growth regulator (0.5 mg·L^−1^ IBA), exhibited no rooting but good growth during the pre-induction period. However, browning and death occurred during the later period. Following treatment with the combination of NAA + IBA, there was an improved effect on rooting induction, and browning was resolved, particularly in the C3 treatment (0.6 mg·L^−1^ NAA + 0.7 mg·L^−1^ IBA), which did not significantly differ from the B1 treatment (*p* < 0.05) but was significantly better than other combinations of different concentrations of plant growth regulators(Figure 2I,J and Figure 4). The exogenous auxin treatment of 0.6 mg·L^−1^ NAA + 0.7 mg·L^−1^ IBA resulted in a 100% rooting rate, with an average of 6.20 roots per plant and an average root length of 4.96 cm. The thick roots had lateral roots, indicating high-quality rooting (Figure 2G–J and Figure 4). Therefore, NAA + IBA was the excellent combination of PGRs for rooting, and the formula suitable for inducing rooting in *M*. ‘Baiyun’ tissue culture was 1/2 MS medium + 15 g·L^−1^ sucrose + 7.0 g·L^−1^ agar + 0.6 mg·L^−1^ NAA + 0.7 mg·L^−1^ IBA.

## 3. Discussion

### 3.1. The Tissue Culture Process of M. ‘Baiyun’ Crabapple Achieves a Well-Established Aseptic System and Excellent Rooting Rate

This study selected mid-March terminal buds as explant material and sterilized them, and compared them to *M. micromalus* Mak. [14], Pingyi sweet tea (*M. hupehensis* Rehd.) [14], Xinjiang wild apple (*M. sieversii* (Ledeb) Roem.) [14], apple (*M. pumila*) rootstocks T337 [15], M26 [16], M9-T337 [16] and other aseptic systems of Malus. Treatment with only 75% alcohol (C_2_H_5_OH) for 20 s significantly reduced the contamination rate by 12.49 times, decreased the dieback rate by 14.02 times, and increased the shoot initiation rate to 90%. This may be because annual buds of the *Malus* and other easily browning plants may carry fewer viruses, pests, and diseases [17]. They also have a strong dividing ability, less phenolic content, and less susceptibility to pathogen invasion, which facilitates establishing an aseptic system for the explant [18]. In addition, we cleaned the explants through ultrasound during the washing stage. Disinfection was environmentally friendly without using highly toxic chemical reagents (HgCl) [19]. At the same time, the innovative cleaning process used a combination of ultrasound and running water, making the explant cleaning more thorough.

The ratio of auxin to cytokinin determines organ differentiation during plant tissue culture. When the auxin–cytokinin ratio is higher, it is excellent for inducting rooting. Conversely, it inducts shoot organs [20,21]. The formulation for inducing rooting in *M.* ‘Baiyun’ tissue culture was 1/2 MS medium with 15 g·L^−1^ sucrose, 7.0 g·L^−1^ agar, 0.6 mg·L^−1^ NAA, and 0.7 mg·L^−1^ IBA, which achieved excellent rooting with a rooting rate of 100% and an average number of roots of 6.20, with an average root length of 4.96 cm. Little time and a single induction can achieve an excellent rooting rate, compared with the induction rooting of other trees such as *M. pumila* [16] and *Prunus armeniaca* L. [22], which minimizes the risk of contamination damage in the rooting process. This is due to more inducing processes that may lead to an increasing risk of plant tissue damage, which may lead to promoting phenolic content to exacerbate browning and affect rooting [23]. Concurrently, the PVP may play an important role in promoting rooting during the induction process [24]. Furthermore, we selected strong dividing ability terminal buds in mid-March as explant material and adopted the plant organ to induct adventitious shoots and roots, which are fine factors to result in an excellent rooting rate [25]. Additionally, the existing techniques for tissue culture of *Malus* are mainly focused on the original species and *M. pumila* Mill [14,15,16]. Therefore, this study creatively proposes tissue culture protocols for a well-established aseptic system and excellent rooting rate for the new cultivars of *M.* ‘Baiyun’.

### 3.2. Effect of Different Asexual Reproduction Techniques on Plant Resistance

Asexual reproduction includes cutting propagation, grafting propagation and plant tissue culture [9,10,26]. Ornamental crabapple cutting propagation is faced with slow rooting, roots that easily rot, difficulty in rooting, plantlets that cannot remove viruses, and other difficulties, while resistance to disease is poor [27]. Grafting propagation is greatly affected by rootstocks. Common rootstocks used in ornamental crabapples are *M*. ‘Dolgo’, *M. hupehensis* and *M. robusta* [3,4,5]. *M*. ‘Dolgo’ was introduced in North America [28]. As a consequence of the impact of climate change, soil, and other environmental differences between North America and China, the characteristics of resistance in China are significantly inferior to those of breeding areas [4,11]. *M. hupehensis* generally has poor flooding tolerance, which is not conducive to its application in the monsoon climate with a rainy summer in south China [29]. The seedlings of *M. robusta* exhibit considerable variation and differentiation, with a lack of consistency in resistance performance [30]. Tissue culture technology can cultivate virus-free and low-variation plantlets and stable resistance characteristics [15,16]. In this study, *M.* ‘Baiyun’, a highly resistant cultivar, has a high rooting rate, spends less time producing and keeps stable resistance performance through tissue culture, which addresses the issue of the highly resistant cultivars not keeping their stable resistance through grafting.

Due to time and geographical constraints, only the terminal buds and stem segments with one axillary bud were available as explant materials. Therefore, there is a lack of research on the tissue culture of other parts of explants, such as embryo, cotyledon, leaf, petiole, flower, etc., necessary to conduct relevant research exploration and perfect the related tissue culture fast propagation systems. Meanwhile, the critical steps in the tissue culture process should be improved, and the problems of yellowing, browning and other problems should be deeply investigated. In addition, as a woody ornamental, crabapple has a low efficiency of stable transformation, and it is not yet practical for most laboratories to produce large quantities of transgenic *Malus* [31]. However, tissue culture plantlets can be used as good transgenic materials [31,32]. Furthermore, ornamental crabapples have different flower, leaf and fruit colors, so it is possible to carry out explorations of related functional genes and improve tissue culture and transgenic technologies so that the functions of crabapple-related genes and their transgenic breeding may be verified in the future.

## 4. Materials and Methods

### 4.1. Plant Materials

*Malus* ‘Baiyun’ (registration no. 20210210) is an excellent flower and fruit cultivar bred at the National Crabapple Germplasm Genetic Center (Yangzhou City, lat. 32°42′ N, long. 119°55′ E, hardiness zone 8), derived from seeds of *Malus* spp., which were collected from Yangzhou, Jiangsu, China in Fall 2011. Subsequently, an individual plant with middle buds opened with single, extremely white flowers and bright red pomes was observed in 2014 and selected for further evaluation (Figure 1A–C and Figure 2A). Its leaves are alternate and ovate (length, 6.8–7.3 cm; width, 3.8–4.2 cm) (Figure 1D). The initial bloom (10% flowers open) of ‘Baiyun’ was in early April in Yangzhou, Jiangsu, China, which is considered midseason flowering among existing crabapple cultivars. ‘Baiyun’ had many fruits. The mature pomes are red, glossy waxy, and have a light yellow flesh (Figure 1C). Fruits are oblate and relatively small (transverse diameter, 1.0–1.3 cm) with persistent sepals from time to time. The fruit persists until late October in Yangzhou, Jiangsu, China. From 2015 to 2017, the plant consistently exhibited 5 petals per flower and white flowers. We named this individual plant ‘Baiyun’.

The initial explants were selected, robust one-year live seedlings of *M.* ‘Baiyun’ shoots that were free of pests and diseases, by grafting and cultivated in a nursery at the National Germplasm Repository of Crabapple at the Nanjing Forestry University (Yangzhou, China; 32°18′~32°48′ N, 119°27′~119°54′ E) (Figure 2A). Stem segments with buds were collected as experimental explants (Figure 2B). The experiment was conducted in the tissue culture laboratory of the College of Forestry and Herbology, Nanjing Forestry University.

### 4.2. Medium and Growth Condition

The medium was prepared according to the two-factor experimental design of inducted shoot proliferation and rooting by adding different concentrations of plant growth regulators (NAA, IBA, 6-BA), agar, sucrose, and an anti-browning agent. The pH value of the medium was adjusted to 5.8–6.0 with 1 mol/L HCl or NaOH. The medium was then boiled, and the agar was melted before being dispensed into wide-mouthed culture bottles. The glass flasks were sealed with gas-permeable plastic film. The glass flasks were placed in an autoclave and sterilized at 121 °C for 20 min [33,34]. Then, the bottle containing the culture medium was transferred to the designated culture room and allowed to cool and solidify before use [35].

### 4.3. Establishment of an Aseptic Culture System

To determine the appropriate time and site for material collection, we collected apical shoots and stem segments with one axillary bud from annual shoots of *M.* ‘Baiyun’ free from pests and diseases (Figure 2B). The explants were collected on the 15th of each month from March to July 2022. The explants were soaked in sterile distilled water ultrasonic bath for 10 min, rinsed with running tap water for 2 h, treated with 75% alcohol (C_2_H_5_OH) for 20 s, washed three times with sterile water and inoculated on MS medium containing 1.0 mg·L^−1^ 6-BA, 0.1 mg·L^−1^ NAA, 30 g·L^−1^ sucrose, 7.0 g·L^−1^ agar, and 2.0 g·L^−1^ PVP [36,37]. Each glass flask was inoculated with one explant, and the experiment was repeated thrice with 10 glass flasks inoculated each time. The growth was observed every 3 d, and after 30 d of culture, the contamination rate (number of contaminated explants/number of inoculated explants × 100%), dieback rate (number of deaths of uncontaminated explants/number of inoculated explants × 100%), and shoot initiation rate (explants with shoots/number of inoculated explants × 100%) were recorded [35,38].

### 4.4. Differentiation and Proliferation of Adventitious Shoots

The *M*. ‘Baiyun’ material was inserted in MS medium to induce adventitious shoots, adding 30 g·L^−1^ sucrose, 0.5 g·L^−1^ PVP, and 7.0 g·L^−1^ agar (Figure 2C,D). Table 1 shows the different concentrations of plant growth regulators used, including 6-BA (0.5 mg·L^−1^, 1.0 mg·L^−1^, 1.5 mg·L^−1^, 2.0 mg·L^−1^), NAA (0.05 mg·L^−1^, 0.1 mg·L^−1^, 0.2 mg·L^−1^, 0.3 mg·L^−1^), and IBA (0.05 mg·L^−1^, 0.1 mg·L^−1^, 0.3 mg·L^−1^, 0.5 mg·L^−1^) and their combinations [39,40]. Explants were also placed on MS medium without PGRs as a control. Each glass flask was inoculated with 3 explants, and this process was repeated 3 times. A total of 10 glass flasks were inoculated each time, and the growth was observed every 3 d. After 30 d of culture, the proliferation coefficients (number of shoots greater than 0.5 cm in height at the end of the proliferation cycle/number of shoots at the beginning of the proliferation cycle) and growth status of the test-tube plantlets were calculated [41].

### 4.5. Differentiation of Rooting

The basic medium was 1/2 MS with 15 g·L^−1^ sucrose and 7.0 g·L^−1^ agar. Plant growth regulators of NAA and IBA were selected, and three gradients were set at 0.5 mg·L^−1^, 1.0 mg·L^−1^, and 1.5 mg·L^−1^. In order to determine the best conditions for rooting proliferation, ten different culture combinations were tested. The PGRs including NAA (0.4 mg·L^−1^, 0.5 mg·L^−1^, 0.6 mg·L^−1^, 1.0 mg·L^−1^, 1.5 mg·L^−1^) and IBA (0.5 mg·L^−1^, 0.7 mg·L^−1^, 1.0 mg·L^−1^, 1.5 mg·L^−1^) [42,43]. 1/2 MS medium without PGRs was used as a control. (Table 2). Each glass flask was inoculated with one explant, and each treatment was repeated three times. Ten glass flasks were inoculated each time, and the growth was observed every 3 d. After 30 d of incubation, the rooting rate, the number of roots, and the average root length were calculated [41,44].

### 4.6. Data Analysis

SPSS 24.0 (IBM Corp., Armonk, NY, USA) was used to perform a one-way analysis of variance (ANOVA) using Duncan’s post hoc method. Data were organized using Microsoft Office Excel 2019 (Microsoft Corp., Redmond, WA, USA). Figures were illustrated using Origin 2019 (Origin Lab Corp., Northampton, MA, USA).

## 5. Conclusions

The best explant material for *M.* ‘Baiyun’ tissue culture was the terminal buds that emerged in mid-March, which were treated with 75% alcohol (C_2_H_5_OH) for 20 s and then washed three times with sterile water. The suitable culture formula for *M.* ‘Baiyun’ adventitious bud induction was MS medium + 30 g·L^−1^ sucrose + 0.5 g·L^−1^ PVP + 7.0 g·L^−1^ agar + 0.5 mg·L^−1^ 6-BA + 0.05 mg·L^−1^ IBA. The formula suitable for inducing rooting in *M*. ‘Baiyun’ tissue culture was 1/2 MS medium + 15 g·L^−1^ sucrose + 7.0 g·L^−1^ agar + 0.6 mg·L^−1^ NAA + 0.7 mg·L^−1^ IBA. Thus, this study has established a shoot proliferation and rooting procedure for the fast propagation of *M.* ‘Baiyun’. Meanwhile, our study is the first to establish an effective organogenesis protocol for new crabapple cultivars using stem segments. The established technology system can be a reference for maintaining and promoting good resistance in new cultivars, researching gene function and the factory nursery of *M.* ‘Baiyun’ or other crabapple species.

## Figures and Tables

**Table 1:** Proliferation of *M*. ‘Baiyun’ shoots after 30 d of culture at different PGRs conditions.

Treatment No.	Plant Growth Regulators and Concentration			Proliferation Coefficient
	6-BA (mg·L^−1^)	NAA (mg·L^−1^)	IBA (mg·L^−1^)	
A1	0.5	0.05	0	1.57 ± 0.25 fgh
A2	0.5	0.1	0	2.80 ± 0.50 c
A3	0.5	0.3	0	1.43 ± 0.15 fgh
A4	1	0.05	0	1.80 ± 0.10 efg
A5	1	0.1	0	3.80 ± 0.20 b
A6	1	0.3	0	1.67 ± 0.21 fgh
A7	2	0.05	0	1.27 ± 0.12 gh
A8	2	0.1	0	1.63 ± 0.32 fgh
A9	2	0.3	0	1.13 ± 0.15 h
A10	0.5	0	0.05	6.27 ± 0.38 a
A11	0.5	0	0.1	2.63 ± 0.51 c
A12	0.5	0	0.3	1.97 ± 0.12 def
A13	1	0	0.05	2.43 ± 0.21 cd
A14	1	0	0.1	2.30 ± 0.53 cde
A15	1	0	0.3	1.97 ± 0.15 def
A16	2	0	0.05	1.63 ± 0.21 fgh
A17	2	0	0.1	1.53 ± 0.15 fgh
A18	2	0	0.3	1.50 ± 0.10 fgh
CK	0	0	0	1.00 ± 0.00 h

Means ± SD with different letters in the columns differ significantly by Duncan’s test (*p* ≤ 0.05).

**Table 2:** Experimental design of single exogenous auxin on rooting.

Auxin (mg·L^−1^)	Treatment No.										
	CK	B1	B2	B3	B4	B5	B6	C1	C2	C3	C4
NAA	0	0.5	1.0	1.5	0	0	0	0.4	0.4	0.6	0.6
IBA	0	0	0	0	0.5	1.0	1.5	0.7	1.0	0.7	1.0

## Data Availability

Data available on request from the authors.

## Abbreviations

NAA	Naphtha acetic acid
6-BA	6-Benzyladenim
PGRs	Plant growth regulators
IBA	3-Indolebutyric acid
ANOVA	Analysis of variance
MS	Murashige and Skoog (1962) medium
PVP	Polyvinyl pyrrolidone
